# Effects of Chronic Sleep Restriction during Early Adolescence on the Adult Pattern of Connectivity of Mouse Secondary Motor Cortex^1^,^2^,^3^

**DOI:** 10.1523/ENEURO.0053-16.2016

**Published:** 2016-06-20

**Authors:** Yazan N. Billeh, Alexander V. Rodriguez, Michele Bellesi, Amy Bernard, Luisa de Vivo, Chadd M. Funk, Julie Harris, Sakiko Honjoh, Stefan Mihalas, Lydia Ng, Christof Koch, Chiara Cirelli, Giulio Tononi

**Affiliations:** 1 Computation and Neural Systems Program, California Institute of Technology, Pasadena, California 91125; 2 Department of Psychiatry, University of Wisconsin-Madison, Madison, Wisconsin 53719; 3 Neuroscience Training Program, University of Wisconsin-Madison, Madison, Wisconsin 53705; 4 Department of Experimental and Clinical Medicine, Section of Neuroscience and Cell Biology, Università Politecnica delle Marche, Torrette di Ancona, Ancona, Italy 60020; 5 Allen Institute for Brain Science, Seattle, Washington 98109; 6 Medical Scientist Training Program, University of Wisconsin-Madison, Health Sciences Learning Center, Madison, Wisconsin 53705

**Keywords:** adolescence, sensitive period, secondary motor cortex, sleep loss

## Abstract

Cortical circuits mature in stages, from early synaptogenesis and synaptic pruning to late synaptic refinement, resulting in the adult anatomical connection matrix. Because the mature matrix is largely fixed, genetic or environmental factors interfering with its establishment can have irreversible effects. Sleep disruption is rarely considered among those factors, and previous studies have focused on very young animals and the acute effects of sleep deprivation on neuronal morphology and cortical plasticity. Adolescence is a sensitive time for brain remodeling, yet whether chronic sleep restriction (CSR) during adolescence has long-term effects on brain connectivity remains unclear. We used viral-mediated axonal labeling and serial two-photon tomography to measure brain-wide projections from secondary motor cortex (MOs), a high-order area with diffuse projections. For each MOs target, we calculated the projection fraction, a combined measure of passing fibers and axonal terminals normalized for the size of each target. We found no homogeneous differences in MOs projection fraction between mice subjected to 5 days of CSR during early adolescence (P25–P30, ≥50% decrease in daily sleep, *n*=14) and siblings that slept undisturbed (*n*=14). Machine learning algorithms, however, classified animals at significantly above chance levels, indicating that differences between the two groups exist, but are subtle and heterogeneous. Thus, sleep disruption in early adolescence may affect adult brain connectivity. However, because our method relies on a global measure of projection density and was not previously used to measure connectivity changes due to behavioral manipulations, definitive conclusions on the long-term structural effects of early CSR require additional experiments.

## Significance Statement

Adolescence is a sensitive period of intense brain remodeling but it is unknown whether chronic disruption of sleep at this age has long-term structural effects on neural circuits. We measured the density of projections between the mouse secondary motor cortex and the rest of the brain, using viral-mediated axonal labeling followed by serial two-photon tomography. Mice underwent 5 days of chronic sleep restriction during early adolescence or slept undisturbed, and brain connectivity was assessed after the mice reached adulthood. The two groups did not differ in any global or homogeneous way. However, machine learning classification performance allows us to conclude that intricate and local heterogeneous changes do persist in adulthood due to chronic sleep restriction.

## Introduction

From early development to the end of adolescence, cortical circuits mature in stages, from early massive synaptogenesis and synaptic pruning, which result in large changes in the absolute number of synapses, to late synaptic refinement, when the initially homogeneous connectivity is reorganized without major changes in synaptic density (Innocenti and Price, 2005; Sanes and Yamagata, 2009; Tau and Peterson, 2010; Uddin et al., 2010). The end result of these processes is the adult anatomical connection matrix. Because this matrix is largely fixed, genetic or environmental factors that interfere with its establishment during development can have irreversible effects. Sleep disruption is rarely considered among these factors, perhaps because one can always sleep longer or deeper at a later time. Thus, few studies have tested the hypothesis that sleep disruption during development may impair the maturation and maintenance of brain circuits (Roffwarg et al., 1966). For example, early experiments used drugs to disturb neonatal sleep, and found long-term neurochemical and behavioral effects, for instance on anxious behavior (for review, see Frank, 2011). However, these changes were likely caused not only by sleep loss, but also by other effects of the drugs used to enforce wake, many of which affect monoaminergic transmission (Frank, 2011). More recent experiments in kittens combined monocular deprivation with 1 week of rapid eye movement (REM) sleep deprivation before the end of the critical period and found a decrease in the size of neurons in the lateral geniculate nucleus of the thalamus (Shaffery et al., 1998), and similar results were obtained after total sleep deprivation (Pompeiano et al., 1995). Chronic REM sleep deprivation alone also leads to the persistence of an immature form of synaptic potentiation in primary visual cortex, suggesting that sleep loss slows down the maturation of cortical circuits (Roffwarg et al., 1966; Shaffery et al., 2002, 2012). Other studies in kittens found that a few hours of total sleep deprivation can immediately impair ocular dominance plasticity when sleep is prevented at the height of the critical period (Frank et al., 2001; Frank, 2011), and acute sleep deprivation in adolescent mice impairs the growth and maintenance of a subset of cortical spines formed after learning (Yang et al., 2014). Most of these experiments focused on preadolescent animals, and morphological and electrophysiological effects were assessed immediately or soon after the end of sleep deprivation. Thus, whether sleep loss during development leaves permanent structural changes in the adult brain was unknown, and even less known were the consequences of sustained sleep disruption during the sensitive period of adolescence (Paus et al., 2008).

Here we tested in mice whether the occurrence of chronic sleep restriction (CSR) during early adolescence has long-term effects on the adult anatomical connection matrix. In rodents, adolescence can be broadly defined as the period from weaning at postnatal day (P)21 to sexual maturity (∼P50–P60; Spear, 2000). Massive synaptogenesis and synaptic pruning occur mainly during the second postnatal week (Aghajanian and Bloom, 1967; Koester and O'Leary, 1992; Micheva and Beaulieu, 1996; De Felipe et al., 1997; Maravall et al., 2004; Ashby and Isaac, 2011; Romand et al., 2011). Synaptic refinement follows in the third and fourth postnatal week, when the initially homogeneous connectivity is reorganized without major changes in synaptic density, and the functional optimization of cortical circuits continues throughout adolescence (Zhang et al., 2002; Cancedda et al., 2004; Seelke et al., 2012; Ko et al., 2013). Thus, during early adolescence (∼P21–P34) the anatomical connection matrix is still being refined. During the same time electroencephalographic (EEG) patterns across the sleep/wake cycle are similar to those seen in adults, and so are total daily sleep amounts ((Gramsbergen, 1976; Frank and Heller, 1997; Nelson et al., 2013); see Materials and Methods for details).

## Materials and Methods

### Animals

**Figure 1.**
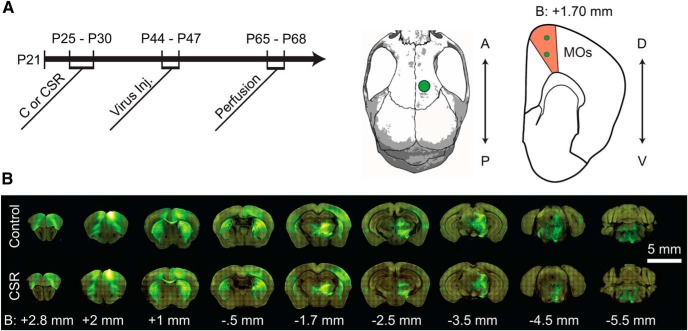
Experimental Timeline and MOs Projections. ***A***, Experimental timeline. Between P25 and P30, mice were allowed to sleep normally (C) or subjected to CSR. All mice were injected with AAV-GFP between P44 and P47, and each mouse was perfused exactly 3 weeks later. Middle, Right, The location of the viral injections on the skull and in a coronal brain section. A, Anterior; P, posterior; D, dorsal; V ventral; B, bregma. ***B***, Example of projections from MOs in two representative mice (C and CSR) 3 weeks after injection of AAV-GFP. Measurements are given in millimeters from bregma. Final analysis included 14 mice in each group.

Five litters of C57BL/6J mice of the same age (*n*=32) were used in one single experiment that included 5 days of chronic sleep restriction (or sleep *ad libitum*) between P25 and P30, surgery for cortical injection of viral tracer at P44–P47, and perfusion for brain collection at P65–P68 (Fig. 1*a*). All animal procedures followed the National Institutes of Health *Guide for the Care and Use of Laboratory Animals* and facilities were reviewed and approved by the IACUC of the University of Wisconsin-Madison, and were inspected and accredited by AAALAC.

### Experimental procedure

At P21 mice were weaned, weighed, and housed in groups (4 per cage) in environmentally controlled conditions (12 h light/dark cycle; lights on at 8:00 A.M., room temperature 23 ± 1°C). At P24 body weight was rechecked and two groups of 16 animals, weight-balanced and sex-balanced, were created from the total pool of 32 mice. Each group was moved into a large cage (60 × 60 × 40 cm) where mice were free to interact. Food and water were provided *ab libitum* and replaced daily at 8 A.M. At P25, the control group was left undisturbed and video-monitored for 5 days, whereas the second group was subjected to 5 days of CSR starting at 8 A.M. At that time adolescent mice show EEG patterns across the sleep/wake cycle similar to those of adult mice, with low-voltage fast activity during wake and REM sleep and large slow waves during NREM sleep (Gramsbergen, 1976; Frank and Heller, 1997). Total daily sleep amounts in young adolescent mice are also at adult levels (Nelson et al., 2013). On the other hand, REM sleep in mice continues to decline during early adolescence, and sleep deprivation is followed by an increase in sleep duration but not in sleep intensity, suggesting that the mechanisms of homeostatic sleep regulation are not fully mature (Nelson et al., 2013).

CSR was enforced using multiple strategies to disrupt sleep. During the day, ecologically relevant stimuli were selected and presented to mice, including continuous exposure to novel objects, changes of cage and bedding, social interaction, and free access to multiple running wheels. Mild forced locomotion on a slowly rotating platform was used to restrict sleep during some parts of the night. The platform was located above a tray filled with 2–3 cm of water, and the rotation speed was low enough that mice could easily avoid falling into the water as long as they moved continuously. Heat lamps were placed ∼2 m above the platform to keep mice at the proper temperature. Video cameras and/or direct visual observation were used to monitor the mice at all times. Several mice were placed on the platform at the same time, and we estimate that each mouse fell into the water no more than 5 times per hour. If a mouse fell often enough such that it did not have a chance to dry, it was removed to a cage filled with novel objects and allowed to dry before being placed back onto the rotating platform. A previous CSR study that lasted 4 days (P25–P29) and used mice implanted with EEG electrodes found that total sleep time throughout the experiment was decreased by ∼70% (de Vivo et al., 2016). After CSR (or sleep *ad libitum*) mice were returned in their home cages (4 per cage), and continued to have access to novel objects (new sets of objects every morning) and running wheels until the end of the experiment at P65–P68. All mice gained weight between P21 (weaning day) and P30 (end of CSR), but controls did so more than CSR mice (C: +44.2 ± 12.2%; CSR: +20.7 ± 9.3%; *t* test, *p*<0.0001), and in each group males grew more than females (C/F +34.8 ± 12.7%; C/M +51.1 ± 12.2%; *t* test, *p* = 0.007; CSR/F +15.8 ±9.8%; CSR/M +27.9 ±8.3%; *t* test, *p*=0.008).

### Stereotaxic injection of AAV for anterograde axonal tracing

Surgery occurred over the course of 4 days (8 mice/day) between P44 and P47. Anterograde axonal tracing from MOs was performed by injecting AAV1.hSyn.eGFP.WPRE.bGH (1.79 × 10^13^ GC/mL) at two different depths using iontophoresis, which allows for small, focal injections (Harris et al., 2012). The day before surgery, glass capillary tubing was heat-pulled to create pipet tips that were then cut and verified under a microscope to obtain tip widths of 10–30 μm. Just prior to surgery, these pipets were filled with virus using capillary action to prevent formation of bubbles. Mice were anesthetized under 2% isoflurane and maintained at 1–2% isoflurane for the duration of surgery. Using sterile technique, mice were fitted into a stereotaxic frame and an incision was made to expose the skull. The skull was cleaned with saline and hydrogen peroxide, and a small burr hole was made in the skull using a dental drill. Any exposed brain was kept moist by saline at all times. The filled pipet was then prepared by lowering a silver wire into the pipet until it contacted virus. An electrical lead was attached to the silver wire, and an electrical ground was connected to a metal clip placed on the skin near the skull. The pipet was then lowered to the surface of the brain 1.7 mm anterior and 0.75 mm lateral (right) from bregma. From the cortical surface, the pipet tip was lowered through the dura and into the brain 0.4 mm. A pause of 2 min was given to allow for a weak seal to form between the brain and the glass of the pipet. Current was then delivered through the pipet tip at 3 μA, alternating 7 s on and 7 s off, and repeating for 5 min to inject viral particles. The pipet tip was then lowered another 0.4 mm (to a total depth of 0.8 mm from the surface of the cortex) and the 5 min current delivery was repeated. After the current was stopped, the pipet tip was kept in place 5 additional minutes to allow for any pressure to dissipate before removal of the tip. After removal of the tip, the incision was sealed using Vetbond, antibiotic gel was applied to the surgical site, and mice were removed from isoflurane. Mice were monitored daily for 7 days following surgery to ensure normal recovery. Two control mice experienced health issues in this period and were killed.

### Perfusion

Three weeks after surgery (P65–P68), mice (16 CSR and 14 controls) were deeply anaesthetized with isoflurane (3% volume) and perfused transcardially with a flush (∼30 s) of saline followed by 4% paraformaldehyde (PFA) in phosphate buffer (PB). Mice were then decapitated, and heads were kept in 4% PFA until shipping to the Allen Institute for serial two-photon tomography.

### Serial two-photon tomography

Briefly, carefully dissected brains were prepared for serial two-photon (STP) tomography, which integrates optical imaging and vibratome sectioning, by first rinsing with PBS before embedding in an agarose block as previously described in detail (Oh et al., 2014). Images were acquired on TissueCyte 1000 2-photon systems (TissueVision) coupled with Mai Tai HP DeepSee lasers (Spectra Physics) using 925 nm wavelength light through a Zeiss 20× water immersion objective (NA = 1.0). One optical plane was imaged 75 µm below the cutting surface. After an entire section was imaged at an XY resolution of ∼0.35 µm/pixel, a 100 µm section was cut by the vibratome and then the specimen was returned to the objective for imaging of the next plane. Images from 140 coronal sections were collected to cover the full mouse brain. Data from one CSR mouse could not be used due to a problem in image alignment. Another CSR mouse was excluded due to problems during imaging, and thus the final analysis included 14 controls and 14 CSR mice.

### Image data processing

The Allen Institute informatics data pipeline managed processing and organization of the images and quantified data for analyses. Algorithms developed for the *Allen Mouse Connectivity Atlas* for signal detection and image registration were used on this dataset (Oh et al., 2014). Detailed descriptions of the neuroinformatics developed for segmentation and registration for this atlas were published recently (Kuan et al., 2015). Briefly, the signal detection algorithm was applied to each image to segment positive fluorescent signals from background. Steps include low-pass filtering to remove noise, followed by adaptive edge/line detection and classification, then integration of the detected results and rejection of artifacts or outliers. For registration, as STP tomography results in inherently aligned section images, we can simply stack the section images together to form a coherent reconstructed 3D volume. Each image stack is first registered to an intermediate “template” brain, created by iteratively averaging across ∼1700 brains from the *Allen Mouse Connectivity Atlas*. Registration to this template occurs in two broad steps; global alignment followed by local alignment effected through a coarse-to-fine deformation registration. The final step is then to align to the 3D Allen Mouse Common Coordinate Framework model.

### Project density estimation

After segmentation and registration, signal was quantified for each voxel (10 ×10 × 10 µm) in the reference space and for each structure in the ontology by combining voxels from the same structure in the 3D reference model.

### Thresholding

The regions that had mean projection fractions <0.1% were removed from the comparison analysis because the signal was too weak to be reliable across mice. For the ipsilateral side of the injection, 56 regions were removed (237 remained) and 92 regions (201 remained) for the contralateral side.

### Injection volume normalization

Due to the experimental difficulties in controlling for the injection volume in every animal, we sought to account for the differences by normalizing by a factor proportional to the injection volume. We observed that a direct division of the projection fractions by injection volumes was not suitable and resulted in a negative correlation between total projection fraction and injection volume (not shown). Thus, we proceeded to normalize the data by fitting a power law:

$$\sum PF=A{\left(InjVol\right)}^{n}+B.$$

Where ∑*PF* is the sum of all projection fractions for an animal (after thresholding), *InjVol* is the injection volume, and (*A*, *B*, *n*) are constants. It can be seen that *B* = 0 as there will be no fluorescence signal in the absence of any injection. Taking the logarithm:

log⁡(∑PF)=nlog⁡(InjVol) +log⁡A.

A linear regression fit allowed us to determine that *n* = 0.216 was optimal and hence all the projection fractions were divided by (*InjVol*)^*n*^. Note that once this normalization is done, the projection fraction values are no longer guaranteed to be ≤1.

### General linear model

A general linear model (GLM) was used to control for differences in the centroids of the injections. This was done on the data after having normalized by the injection volume as described above. For every region, a GLM was fit to see the effect of the group type (CSR or control) on the normalized projection fraction. To allow comparisons between regions, every region was normalized to have unit mean. This was followed by fitting the following GLM:

$$PF_{i,j}=\beta_{j}+k_{xj}x_{i}+k_{yj}y_{i}+k_{zj}z_{i}+k_{cj}c_{i}.$$

**Figure 2.**
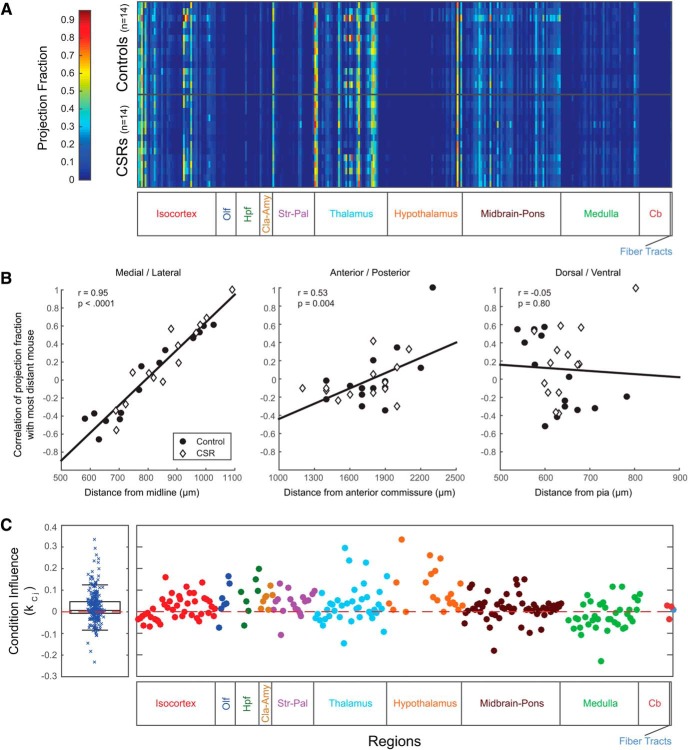
Projection Fractions in Controls and CSR Mice. ***A***, A plot of projection fractions (normalized by injection volume) across all mice (*y*-axis) and all regions (*x*-axis). ***B***, Correlations of projection fraction patterns in mice with injection site. The mouse with the most extreme distance in injection location is taken as the basis for correlation (and is by definition equal to one). All regions were first normalized to have unit means to account for differences between injections. Without this normalization, the mean pairwise correlation of all animals is 0.903 ± 0.07. Distance in micrometers is measured to the center of the injection site from the midline (826 ± 140, left), the midline merging of the anterior commissure (1771 ± 262, middle) or the pial surface (636± 60, right). Values are given for Pearson’s rho. ***C***, Left, Box plot of the condition influence, $k_{cj}$, of the GLM for all regions. A positive $k_{cj}$ indicates that the control group has a higher connectivity than the CSR group (see Results for details). Right, An expanded version that is aligned and color-coded to match the subdivision in major anatomical regions.

Where *PF_i, j_* is the normalized projection fraction for animal *i* at region *j*, *x_i_* is the medial-lateral distance of the injection site from the midline for animal *i* after adjusting for all animals, such that $\overset{¯}{x_{i}}=0$. $y_{i}$ corresponds to the anterior-posterior distance (from the anterior commissure) and $z_{i}$ to depth measurements (from the pia). $c_{i}$ corresponds to the condition of the animal, where control =1 and CSR =-1. The terms $k_{xj}$, $k_{yj}$, $k_{zj}$, and $k_{cj}$ are factors that capture the influence of their corresponding variable and are determined by maximum likelihood estimation with the MATLAB 2015b Statistics and Machine Learning Toolbox. $\beta_{j}$ is the intercept term of the GLM for every region j. It should be noted that if $k_{cj}$ is positive, then that indicates that control animals ($c_{i}=1)$ will have an increased projection fraction due to their condition. The opposite is true if $k_{cj}\;$is negative. If $k_{cj}\;=0$ then there is no effect due to the condition. Although our analysis shows that individually $k_{cj}$ values are not significant, we observe that all of the $k_{cj}$ values are positively biased with a positive mean (Fig. 2*C*). The mean of all $k_{cj}$ values is called $\mu_{k_{c}}.$ However, the $\mu_{k_{c}}>0$ result does not pass a bootstrap significance test as described below.

### Bootstrap test

To test the confidence of the positive $\mu_{k_{c}}$ result, we performed a bootstrap test on the data. Each group was separately resampled (with replacement) to create a total of 50 new data sets while maintaining the same size for each group. Thus, any single resampling case may have some animals selected multiple times and others not selected at all. This allowed for 2500 (50 CSR ×50 Control) comparisons where we determined the fraction of times that $\mu_{k_{c}}$ was negative as the *p* value. Note that every comparison involved determining a new GLM for every region. With these comparisons, the best *p* value that may be claimed is$\;1/50^{2}$ though the attained *p* values were significantly larger. For the test where the female mice were investigated separately, $\mu_{k_{c}}$ changed sign ($\mu_{k_{c}}$ <0) and hence the opposite one-tailed bootstrap was performed (ratio of positive $\mu_{k_{c}}$ to total number of comparisons).

### Animal classification

Machine learning techniques were implemented to classify the animals between CSR and control groups. This was done after fitting a modified GLM as described above that did not include a condition parameter. The dependence of the injection centroid for every animal was then subtracted such that, to the best of our knowledge, only condition was a factor in influencing projection fractions. The algorithms were trained on n-1 animals and classification was tested on the excluded animal. This process was iterated and the classification performance was quantified as the ability to classify all animals in this manner. Using a standard logit-boost decision tree to minimize binomial deviance gave the best classification accuracy at 71% (8 errors). This was due to the large overlap between animal projection values, such that no specific regions were adequate to instantly determine whether there were differences between the two groups. Hence, to improve the performance, a preprocessing step as inspired by role-base similarity was applied (Beguerisse-Díaz et al., 2014). Here, correlations between all regions were calculated to generate a 237×237 correlation matrix (ipsilateral hemisphere only considered). The matrix was thresholded at zero to create a positive-only correlation matrix in addition to zeroing the diagonal. Multiscale clustering was achieved using the dynamics-based clustering framework of Markov Stability (Delvenne et al., 2010; Schaub et al., 2012; Billeh et al., 2014). The clustering algorithm used a Markov process to find clusters at different scales (Schaub et al., 2012). Each cluster was then merged by summing each region within it. For instance, if a group of regions were grouped into a cluster, then for each animal the projection fractions were summed up to attain merged projection values for all regions in that cluster. At the different scales found by the Markov stability algorithm, a binary decision tree that minimizes the binomial deviance between a root node and targets was used to classify the compacted data (MATLAB 2015b Statistics and Machine Learning Toolbox). The classification improved to an accuracy of 82% (5 errors).

### Anatomical analysis of MOs projections

Overall MOs projection pattern was highly consistent with what reported in the literature (Stuesse and Newman, 1990; Reep et al., 2008). Specifically, starting from the injection site in MOs, strong labeling was seen in fiber tracts extending rostrally and bilaterally into MOs, primary motor cortex, orbital area, and claustrum. Fibers weakly labeled were seen extending into the olfactory tubercle and surrounding olfactory areas bilaterally, including tenia tecta. All these bilateral projections were stronger in the hemisphere ipsilateral to the injection. Weak bilateral labeled fibers were also seen along the midline in the lateral septal nucleus and diagonal band nucleus, with roughly equal strength in both hemispheres. Moving caudally from the site of the injection, projections could be seen bilaterally in MOs, primary motor cortex and claustrum, and ipsilaterally in retrosplenial cortex. Ipsilateral primary and secondary somatosensory cortex was strongly labeled, especially in the deep and superficial layers (a similar pattern was seen in contralateral somatosensory cortex, but fluorescence was much weaker). Weak fluorescence was visible ipsilaterally in auditory and visual cortex and in postsubiculum, mostly in the deep layers, with weaker fluorescence in the same areas on the contralateral side. A reliable signal was visible across all layers in bilateral ectorhinal cortex. Fibers descended ventrally and bilaterally into caudate putamen, nucleus accumbens, and basolateral amygdala, as well as in the dorsal portion of the bed nucleus of the stria terminalis. Fibers also descended within the internal capsule toward the thalamus ipsilateral to the injection. Thalamic projections extended into rostral reticular thalamic nucleus, ventral posteromedial nucleus, ventral medial nucleus, and ventral anterior-lateral nucleus of the thalamus. More caudally, projections were visible bilaterally (but always stronger ipsilaterally) in central medial nucleus, ventral medial nucleus, mediodorsal nucleus, parafascicular nucleus, and rhomboid nucleus, whereas ipsilateral projections were present in central lateral nucleus and posterior complex. Very faint projections were sometimes visible in the hippocampus, usually along the medial dorsal border of CA1 and dentate gyrus. Further caudally, ipsilateral projections were seen in the zona incerta, subthalamic nucleus, parasubthalamic nucleus, substantia nigra, ventral tegmental area, and extended dorsally into retrorubral area, red nucleus, midbrain reticular nucleus, and superior colliculus. Much weaker projections were visible in the same areas contralaterally. Fibers were also visible in periaqueductal gray and midline nuclei including Edinger–Westphal nucleus and rostral linear nucleus raphe. Midline nuclei showed similar projection strength bilaterally, and projections to periaqueductal gray were visible bilaterally, although they were stronger ipsilaterally. Additionally, strongly labeled fibers were present in the cerebral peduncle along the ventral side of the brain in the ipsilateral hemisphere. Throughout the pons, diffuse projections were visible in roughly equal strength bilaterally, largely in the pontine reticular nucleus. Major projection fibers descended to the medulla via the pyramidal tract resulting in a similar diffuse projection pattern with bilateral weak projections to vestibular nuclei and stronger projections to ventral midline nuclei including raphe magnus, raphe pallidus, magnocellular reticular nucleus, parapyramidal nucleus, and inferior olivary complex. More caudally through the medulla, ipsilateral fibers begin to move contralaterally as the pyramidal tract decussates. To our knowledge, no projections to the hippocampus from MOs have been previously reported in rats or mice. We noted very faint traces of labeled projections in the most medial dorsal portions of the hippocampus along the borders of CA1. Though faint, these projections were visible in all mice. In general, because we expected that thin, distal projections would be more affected by CSR, we aimed at including as MOs targets all regions that showed labeled projections, even if those projections were sparse. To verify that thin projections were not a result of noise or false positives, and that other sparse projections were not lost to false negatives, we manually inspected positive signal masks generated after signal detection and compared them to fluorescence images in two mice. We found that the detected signal was highly concordant with observed fluorescence of all intensities, with very few false positives or negatives.

### Statistics

Values in the text and figures are reported as mean ± SD. Experiments were analyzed using two-tailed *t* tests, linear regression, a general linear model (see above), bootstrap test (see above), and computation of binomial cumulative distribution function probabilities. All *p* values <0.05 were considered significant *a priori*. Analysis was performed in MATLAB and all statistical tests are summarized in Table 1.

**Table 1.** Statistics

**Property**	**Data Structure**	**Type of test**	***p* value**
Fig 2B: medial–lateral effect on injections	Normally distributed	Student’s *t* test	<0.0001
Fig 2B: anterior–posterior effect on injections	Normally distributed	Student’s *t* test	0.004
Fig 2B: dorsal–ventral effect on injections	Normally distributed	Student’s *t* test	0.796
Control/CSR influence on connectivity ($\mu_{k_{c}}$ >0)	Normality not assumed	One-tailed bootstrap	0.252
Males: control/CSR influence on connectivity ($\mu_{k_{c}}$ >0)	Normality not assumed	One-tailed bootstrap	0.491
Females: control/CSR influence on connectivity ($\mu_{k_{c}}$ <0)	Normality not assumed	One-tailed bootstrap	0.258
Control/CSR influence on weight (control > CSR)	Normally distributed	Paired *t* test	<0.0001
Control: male/female weight gain (male > female)	Normally distributed	Paired *t* test	0.007
CSR: male/female weight gain (male > female)Accuracy of classification algorithm	Normally distributedBinomial distributed	Paired *t* testBinomial cumulative distribution function test	0.0080.0005

## Results

Mice of the same age from five litters were split into two groups (Fig. 1*A*). One group was subjected to 5 days of CSR in the middle of early adolescence, from P25 to P30, using ecologically relevant stimuli (see Materials and Methods), whereas during the same period control siblings were allowed to sleep *ad libitum*. At the beginning of the experiment, each group included 16 mice, but two animals in each condition were excluded for various technical reasons (see Materials and Methods). The final analysis therefore included 14 controls and 14 CSR mice. Mice were not equipped with EEG electrodes to avoid potential damage to the cortex. However, based on continuous visual monitoring and a previous study with EEG recordings using similar sleep restriction methods (de Vivo et al., 2016), we estimate that overall sleep loss was between 50 and 60% (see Materials and Methods). Approximately 2 weeks later (P44–P47) CSR mice and controls were injected with recombinant adeno-associated virus (AAV)-expressing enhanced green fluorescent protein (EGFP) in the right MOs, to map its projections. We focused on MOs because it has diffuse projections (Zingg et al., 2014) and is highly plastic (Cao et al., 2015). Exactly 3 weeks after each animal’s injection the brains were perfused. Fluorescent signals were then imaged using serial two-photon tomography and informatically reconstructed within the Allen Mouse Common Coordinate Framework, a high-resolution coordinate system that allows the systematic analysis of the entire brain (see Materials and Methods; Oh et al., 2014).

As expected, robust anterograde tracing from right MOs was observed throughout the brain (Fig. 1*B*). The overall pattern of projections was consistent across mice and similar to the one previously described for rat supplementary motor cortex, also known as medial agranular cortex (Stuesse and Newman, 1990; Reep et al., 2008). Briefly, strong projections were seen to orbital area, primary motor cortex, primary and secondary somatosensory cortex, claustrum, striatum, many thalamic nuclei, as well as zona incerta, ventral tegmental area, midbrain reticular nucleus, and several midline nuclei in the pons (see Materials and Methods for detailed anatomical description). Projections were always stronger, or only present, on the side of the injection, again consistent with previous studies showing that most MOs projections are ipsilateral (Stuesse and Newman, 1990; Reep et al., 2008). Thus, subsequent analyses primarily focused on the right side.

To identify potential differences in connectivity between CSR mice and controls we examined the projection fraction (also referred to as projection density) values for each brain structure that receives axonal projections from MOs. The projection fraction is defined as the total number of voxels that fluoresce in a target brain structure divided by the total number of voxels in that structure (see Materials and Methods). Hence, projection fraction is positive-only, has a maximal value of 1, and its use allows to control for differences in volume across anatomically defined regions. Note that the projection fraction includes both passing fibers and axon terminals, because they could not be differentiated informatically. Before direct comparisons between the two groups were made, projection fractions were normalized to control for small differences in injection volume across mice (see Materials and Methods). Moreover, regions with very weak signal were removed (projection fractions <0.1%; see Materials and Methods). For the ipsilateral hemisphere, this thresholding resulted in dropping 56 regions from a total of 293, leaving 237 regions for analysis (Table 2). To avoid discarding genuine weak projections, we set the threshold for projection fraction fairly low. To ensure that signal within the weakest of the 237 regions was not simply due to false signal detection, detected signal overlays were compared to raw fluorescent images in two mice. Manual inspection in these mice confirmed that there were very few false positives and negatives, meaning that weak detected signals corresponded closely to real fluorescence. Figure 2*A* visualizes the normalized and thresholded data for the two groups; every row corresponds to a different mouse injection into MOs and every column is a different region. The projection fraction from MOs to that brain region is plotted by color.

**Table 2.** List of brain regions

**Region no.**	**Region abbreviation**	**Region name**	**Region category**
1	FRP	Frontal pole, cerebral cortex	Isocortex
2	MOp	Primary motor area	Isocortex
3	MOs	Secondary motor area	Isocortex
4	SSp-n	Primary somatosensory area, nose	Isocortex
5	SSp-bfd	Primary somatosensory area, barrel field	Isocortex
6	SSp-ll	Primary somatosensory area, lower limb	Isocortex
7	SSp-m	Primary somatosensory area, mouth	Isocortex
8	SSp-ul	Primary somatosensory area, upper limb	Isocortex
9	SSp-tr	Primary somatosensory area, trunk	Isocortex
10	SSp-un	Primary somatosensory area, unassigned	Isocortex
11	SSs	Supplemental somatosensory area	Isocortex
12	GU	Gustatory areas	Isocortex
13	VISC	Visceral area	Isocortex
14	AUDd	Dorsal auditory area	Isocortex
15	AUDp	Primary auditory area	Isocortex
16	AUDpo	Posterior auditory area	Isocortex
17	AUDv	Ventral auditory area	Isocortex
18	VISal	Anterolateral visual area	Isocortex
19	VISam	Anteromedial visual area	Isocortex
20	VISl	Lateral visual area	Isocortex
21	VISp	Primary visual area	Isocortex
22	VISpl	Posterolateral visual area	Isocortex
23	VISpm	Posteromedial visual area	Isocortex
24	VISli		Isocortex
25	VISpor		Isocortex
26	ACAd	Anterior cingulate area, dorsal part	Isocortex
27	ACAv	Anterior cingulate area, ventral part	Isocortex
28	PL	Prelimbic area	Isocortex
29	ILA	Infralimbic area	Isocortex
30	ORBl	Orbital area, lateral part	Isocortex
31	ORBm	Orbital area, medial part	Isocortex
32	ORBvl	Orbital area, ventrolateral part	Isocortex
33	AId	Agranular insular area, dorsal part	Isocortex
34	AIp	Agranular insular area, posterior part	Isocortex
35	AIv	Agranular insular area, ventral part	Isocortex
36	RSPagl	Retrosplenial area, lateral agranular part	Isocortex
37	RSPd	Retrosplenial area, dorsal part	Isocortex
38	RSPv	Retrosplenial area, ventral part	Isocortex
39	VISa		Isocortex
40	VISrl		Isocortex
41	TEa	Temporal association areas	Isocortex
42	PERI	Perirhinal area	Isocortex
43	ECT	Ectorhinal area	Isocortex
45	AOB	Accessory olfactory bulb	Olfactory areas
46	AON	Anterior olfactory nucleus	Olfactory areas
47	TT	Taenia tecta	Olfactory areas
48	DP	Dorsal peduncular area	Olfactory areas
49	PIR	Piriform area	Olfactory areas
50	NLOT	Nucleus of the lateral olfactory tract	Olfactory areas
51	COAa	Cortical amygdalar area, anterior part	Olfactory areas
57	CA3	Field CA3	Hippocampal formation
59	FC	Fasciola cinerea	Hippocampal formation
60	IG	Induseum griseum	Hippocampal formation
61	ENTl	Entorhinal area, lateral part	Hippocampal formation
65	POST	Postsubiculum	Hippocampal formation
66	PRE	Presubiculum	Hippocampal formation
67	SUB	Subiculum	Hippocampal formation
68	CLA	Claustrum	Claustrum + amygdala
69	EPd	Endopiriform nucleus, dorsal part	Claustrum + amygdala
70	EPv	Endopiriform nucleus, ventral part	Claustrum + amygdala
71	LA	Lateral amygdalar nucleus	Claustrum + amygdala

72	BLA	Basolateral amygdalar nucleus	Claustrum + amygdala
73	BMA	Basomedial amygdalar nucleus	Claustrum + amygdala
74	PA	Posterior amygdalar nucleus	Claustrum + amygdala
75	CP	Caudoputamen	Striatum + pallidum
76	ACB	Nucleus accumbens	Striatum + pallidum
77	FS	Fundus of striatum	Striatum + pallidum
78	OT	Olfactory tubercle	Striatum + pallidum
79	LSc	Lateral septal nucleus, caudal (caudodorsal) part	Striatum + pallidum
80	LSr	Lateral septal nucleus, rostral (rostroventral) part	Striatum + pallidum
83	SH	Septohippocampal nucleus	Striatum + pallidum
84	AAA	Anterior amygdalar area	Striatum + pallidum
86	CEA	Central amygdalar nucleus	Striatum + pallidum
87	IA	Intercalated amygdalar nucleus	Striatum + pallidum
88	MEA	Medial amygdalar nucleus	Striatum + pallidum
89	GPe	Globus pallidus, external segment	Striatum + pallidum
90	GPi	Globus pallidus, internal segment	Striatum + pallidum
91	SI	Substantia innominata	Striatum + pallidum
92	MA	Magnocellular nucleus	Striatum + pallidum
94	NDB	Diagonal band nucleus	Striatum + pallidum
96	BST	Bed nuclei of the stria terminalis	Striatum + pallidum
97	BAC	Bed nucleus of the anterior commissure	Striatum + pallidum
98	VAL	Ventral anterior-lateral complex of the thalamus	Thalamus
99	VM	Ventral medial nucleus of the thalamus	Thalamus
100	VPL	Ventral posterolateral nucleus of the thalamus	Thalamus
101	VPLpc	Ventral posterolateral nucleus of the thalamus, parvicellular part	Thalamus
102	VPM	Ventral posteromedial nucleus of the thalamus	Thalamus
103	VPMpc	Ventral posteromedial nucleus of the thalamus, parvicellular part	Thalamus
104	SPFm	Subparafascicular nucleus, magnocellular part	Thalamus
105	SPFp	Subparafascicular nucleus, parvicellular part	Thalamus
106	SPA	Subparafascicular area	Thalamus
107	PP	Peripeduncular nucleus	Thalamus
108	MG	Medial geniculate complex	Thalamus
110	LP	Lateral posterior nucleus of the thalamus	Thalamus
111	PO	Posterior complex of the thalamus	Thalamus
112	POL	Posterior limiting nucleus of the thalamus	Thalamus
113	SGN	Suprageniculate nucleus	Thalamus
114	AV	Anteroventral nucleus of thalamus	Thalamus
115	AM	Anteromedial nucleus	Thalamus
116	AD	Anterodorsal nucleus	Thalamus
117	IAM	Interanteromedial nucleus of the thalamus	Thalamus
118	IAD	Interanterodorsal nucleus of the thalamus	Thalamus
119	LD	Lateral dorsal nucleus of thalamus	Thalamus
120	IMD	Intermediodorsal nucleus of the thalamus	Thalamus
121	MD	Mediodorsal nucleus of thalamus	Thalamus
122	SMT	Submedial nucleus of the thalamus	Thalamus
123	PR	Perireunensis nucleus	Thalamus
124	PVT	Paraventricular nucleus of the thalamus	Thalamus
125	PT	Parataenial nucleus	Thalamus
126	RE	Nucleus of reunions	Thalamus
127	RH	Rhomboid nucleus	Thalamus
128	CM	Central medial nucleus of the thalamus	Thalamus
129	PCN	Paracentral nucleus	Thalamus
130	CL	Central lateral nucleus of the thalamus	Thalamus
131	PF	Parafascicular nucleus	Thalamus
132	RT	Reticular nucleus of the thalamus	Thalamus
133	IGL	Intergeniculate leaflet of the lateral geniculate complex	Thalamus
134	LGv	Ventral part of the lateral geniculate complex	Thalamus
135	SubG	Subgeniculate nucleus	Thalamus
137	LH	Lateral habenula	Thalamus

138	SO	Supraoptic nucleus	Hypothalamus
140	PVH	Paraventricular hypothalamic nucleus	Hypothalamus
142	PVi	Periventricular hypothalamic nucleus, intermediate part	Hypothalamus
145	AVP	Anteroventral preoptic nucleus	Hypothalamus
147	DMH	Dorsomedial nucleus of the hypothalamus	Hypothalamus
158	VLPO	Ventrolateral preoptic nucleus	Hypothalamus
160	LM	Lateral mammillary nucleus	Hypothalamus
161	MM	Medial mammillary nucleus	Hypothalamus
162	SUM	Supramammillary nucleus	Hypothalamus
163	TMd	Tuberomammillary nucleus, dorsal part	Hypothalamus
164	TMv	Tuberomammillary nucleus, ventral part	Hypothalamus
166	PMd	Dorsal premammillary nucleus	Hypothalamus
168	PVHd	Paraventricular hypothalamic nucleus, descending division	Hypothalamus
170	PH	Posterior hypothalamic nucleus	Hypothalamus
171	LHA	Lateral hypothalamic area	Hypothalamus
172	LPO	Lateral preoptic area	Hypothalamus
173	PST	Preparasubthalamic nucleus	Hypothalamus
174	PSTN	Parasubthalamic nucleus	Hypothalamus
176	STN	Subthalamic nucleus	Hypothalamus
177	TU	Tuberal nucleus	Hypothalamus
178	ZI	Zona incerta	Hypothalamus
179	SCs	Superior colliculus, sensory related	Midbrain + pons
180	IC	Inferior colliculus	Midbrain + pons
181	NB	Nucleus of the brachium of the inferior colliculus	Midbrain + pons
182	SAG	Nucleus sagulum	Midbrain + pons
183	PBG	Parabigeminal nucleus	Midbrain + pons
184	MEV	Midbrain trigeminal nucleus	Midbrain + pons
185	SNr	Substantia nigra, reticular part	Midbrain + pons
186	VTA	Ventral tegmental area	Midbrain + pons
187	RR	Midbrain reticular nucleus, retrorubral area	Midbrain + pons
188	MRN	Midbrain reticular nucleus	Midbrain + pons
189	SCm	Superior colliculus, motor related	Midbrain + pons
190	PAG	Periaqueductal gray	Midbrain + pons
191	APN	Anterior pretectal nucleus	Midbrain + pons
192	MPT	Medial pretectal area	Midbrain + pons
193	NOT	Nucleus of the optic tract	Midbrain + pons
194	NPC	Nucleus of the posterior commissure	Midbrain + pons
195	OP	Olivary pretectal nucleus	Midbrain + pons
196	PPT	Posterior pretectal nucleus	Midbrain + pons
197	CUN	Cuneiform nucleus	Midbrain + pons
198	RN	Red nucleus	Midbrain + pons
199	III	Oculomotor nucleus	Midbrain + pons
200	EW	Edinger–Westphal nucleus	Midbrain + pons
201	IV	Trochlear nucleus	Midbrain + pons
202	VTN	Ventral tegmental nucleus	Midbrain + pons
203	AT	Anterior tegmental nucleus	Midbrain + pons
204	LT	Lateral terminal nucleus of the accessory optic tract	Midbrain + pons
205	SNc	Substantia nigra, compact part	Midbrain + pons
206	PPN	Pedunculopontine nucleus	Midbrain + pons
207	IF	Interfascicular nucleus raphe	Midbrain + pons
208	IPN	Interpeduncular nucleus	Midbrain + pons
209	RL	Rostral linear nucleus raphe	Midbrain + pons
210	CLI	Central linear nucleus raphe	Midbrain + pons
211	DR	Dorsal nucleus raphe	Midbrain + pons
212	NLL	Nucleus of the lateral lemniscus	Midbrain + pons
213	PSV	Principal sensory nucleus of the trigeminal	Midbrain + pons
214	PB	Parabrachial nucleus	Midbrain + pons
215	SOC	Superior olivary complex	Midbrain + pons
216	B	Barringtons nucleus	Midbrain + pons

217	DTN	Dorsal tegmental nucleus	Midbrain + pons
218	PCG	Pontine central gray	Midbrain + pons
219	PG	Pontine gray	Midbrain + pons
220	PRNc	Pontine reticular nucleus, caudal part	Midbrain + pons
221	SG	Supragenual nucleus	Midbrain + pons
222	SUT	Supratrigeminal nucleus	Midbrain + pons
223	TRN	Tegmental reticular nucleus	Midbrain + pons
224	V	Motor nucleus of trigeminal	Midbrain + pons
225	CS	Superior central nucleus raphe	Midbrain + pons
226	LC	Locus ceruleus	Midbrain + pons
227	LDT	Laterodorsal tegmental nucleus	Midbrain + pons
228	NI	Nucleus incertus	Midbrain + pons
229	PRNr	Pontine reticular nucleus	Midbrain + pons
230	RPO	Nucleus raphe pontis	Midbrain + pons
231	SLC	Subceruleus nucleus	Midbrain + pons
232	SLD	Sublaterodorsal nucleus	Midbrain + pons
236	CU	Cuneate nucleus	Medulla
239	NTB	Nucleus of the trapezoid body	Medulla
240	NTS	Nucleus of the solitary tract	Medulla
241	SPVC	Spinal nucleus of the trigeminal, caudal part	Medulla
242	SPVI	Spinal nucleus of the trigeminal, interpolar part	Medulla
243	SPVO	Spinal nucleus of the trigeminal, oral part	Medulla
244	VI	Abducens nucleus	Medulla
245	VII	Facial motor nucleus	Medulla
246	ACVII	Accessory facial motor nucleus	Medulla
247	AMB	Nucleus ambiguus	Medulla
248	DMX	Dorsal motor nucleus of the vagus nerve	Medulla
249	GRN	Gigantocellular reticular nucleus	Medulla
250	ICB	Infracerebellar nucleus	Medulla
251	IO	Inferior olivary complex	Medulla
252	IRN	Intermediate reticular nucleus	Medulla
253	ISN	Inferior salivatory nucleus	Medulla
254	LIN	Linear nucleus of the medulla	Medulla
255	LRN	Lateral reticular nucleus	Medulla
256	MARN	Magnocellular reticular nucleus	Medulla
257	MDRNd	Medullary reticular nucleus, dorsal part	Medulla
258	MDRNv	Medullary reticular nucleus, ventral part	Medulla
259	PARN	Parvicellular reticular nucleus	Medulla
260	PAS	Parasolitary nucleus	Medulla
261	PGRNd	Paragigantocellular reticular nucleus, dorsal part	Medulla
262	PGRNl	Paragigantocellular reticular nucleus, lateral part	Medulla
263	NR	Nucleus of Roller	Medulla
264	PRP	Nucleus prepositus	Medulla
265	PPY	Parapyramidal nucleus	Medulla
266	LAV	Lateral vestibular nucleus	Medulla
267	MV	Medial vestibular nucleus	Medulla
268	SPIV	Spinal vestibular nucleus	Medulla
269	SUV	Superior vestibular nucleus	Medulla
270	x	Nucleus x	Medulla
271	XII	Hypoglossal nucleus	Medulla
272	y	Nucleus y	Medulla
273	RM	Nucleus raphe magnus	Medulla
274	RPA	Nucleus raphe pallidus	Medulla
275	RO	Nucleus raphe obscurus	Medulla
290	FN	Fastigial nucleus	Cerebellum
291	IP	Interposed nucleus	Cerebellum
292	DN	Dentate nucleus	Cerebellum
293	fiber tracts	Fiber tracts	Fiber tracts

A challenge in the outlined experiments is the difficulty in precisely replicating the injection site position. To determine the effect of such variations, plots of the mouse projection correlations relative to the mice with the injections farthest from the anatomical landmark for each axis are shown in Figure 2*B*. Note the farthest injected mouse, for instance most distant from the midline, has a perfect correlation of 1 as it is compared with itself. As can be seen there is a strong dependence relative to the injected medial-lateral position (*r* = 0.954). Similar patterns could be observed for the other dimensions (Fig. 2*B*). We emphasize that in determining the correlation for Figure 2*B*, we normalize by the mean of every region (to have unit mean) to account for differences between injections, which is why we observe negative correlation values. Without this normalization and using solely the heat map in Figure 2*A* gives a high mean pairwise correlation between all injections (0.903 ± 0.07). The effect also showed site specificity where, for instance, the anterior–posterior axis had a strong relationship in the isocortex (*r* = 0.602) and a significant influence by the depth axis was seen in the olfactory areas (*r* = 0.340). To account for this experimental variance, a GLM was fit to the normalized unit-mean projection fraction for every region (see Materials and Methods for more details). In the GLM, the effect of condition (Control or CSR) on a specific region is captured by a parameter $k_{cj}$, where, by construction, if $k_{cj}$ is positive then the control group has a higher projection fraction to region j relative to the CSR group and vice versa. We found that $k_{cj}$ was not significant on a region level, though the distribution of $k_{cj}$ values did appear more positively biased with a positive mean (Fig. 2*C*, left). However, running a bootstrap to test this effect yielded nonsignificant results ($\mu_{k_{c}}=0.026$, p=0.252; see Materials and Methods). Observing the distribution of $k_{c}$ across the different macro-regions (Fig. 2*C*, right) indicates that certain brain regions may be more affected than others by sleep restriction. Once again, however, none of the divisions considered passed the bootstrap significance test. Performing the same analysis on the contralateral side yielded similar results.

To determine whether there were sex-specific differences, we performed the same analysis on the males and female animals separately. This was possible as we had a similar number of males and females (14 males, 8 controls; 15 females, 6 controls). Our results show that the sleep deprivation paradigm did not influence the males’ ($\mu_{k_{c}}=0.013$, p=0.491) nor the females’ ($\mu_{k_{c}}=-0.034$, p=0.258) mesoscale connectivity. Due to the small number of animals, however, we cannot rule out that subtle effects do exist that we are unable to detect.

We also investigated whether we could use the MOs normalized projection fractions (adjusted for injection positions; see Materials and Methods) to classify animals using machine-learning techniques. We trained classification algorithms on all but one animal, used the algorithm to predict the group of the excluded mouse, and repeated the procedure for all mice. We then evaluated the performance of our algorithm by its ability to classify all 28 mice. The best performance we could attain on the normalized data was 71% (8 errors; see Materials and Methods for details). To see if we could improve classification accuracy, we applied a preprocessing step as inspired by a newly developed graph-theory technique termed role-base similarity (Beguerisse-Díaz et al., 2014; see Materials and Methods). Briefly, we found the positive correlations between all regions to create a positive-only correlation matrix that was then clustered at different levels of granularity using a Markov stability algorithm (Delvenne et al., 2010; Schaub et al., 2012; Billeh et al., 2014). By considering different levels of granularity and using classification tree algorithms on the compacted data, we reached a classification accuracy of 82% (5 errors; see Materials and Methods). Because the classification problem is binomial in nature, the classification accuracy corresponds to a *p* value of 0.0005 (determined from a binomial cumulative distribution function with 5 errors, 28 attempts, at a probability of 0.5). This indicates that although CSR does not affect the brain in a single homogenous direction, it does have an intricate heterogeneous effect that can be captured by machine learning classification. Overall, the variability observed in the decision trees from dropping animals did not show a clear hypothesis for *post hoc* testing. Nonetheless, we conclude that long-term changes in brain connectivity at the mesoscopic level do occur, and further investigations are required to fully uncover the differences.

## Discussion

To our knowledge, this is the first study that tested whether there are structural changes in the adult mammalian brain after sleep was restricted during early adolescence. Brains were collected soon after mice reached adulthood, but younger mice were not tested. Thus, there may have been acute effects of CSR that we missed. Our goal, however, was to search for late, possibly irreversible effects of sleep loss on the adult connectivity. We found some evidence that early adolescence may affect the adult brain connectivity, but the changes were subtle and heterogeneous. This finding may be a true biological result, and/or it may reflect the technical limitations of our approach. The method implemented here was never used before to compare projection strength across animals in response to a behavioral manipulation. Moreover, it is based on a measure of “projection density” that combines both fibers of passage and axonal terminals and thus specific effects on the terminals may have been missed and may be better assessed using array tomography combined with excitatory and inhibitory presynaptic and postsynaptic markers (Wang et al., 2014). We note, however, that the method was sensitive enough to be significantly affected by small changes in the injection site along the medial–lateral or anterior–posterior axes, which led in some cases to noticeable differences in projection fraction profiles across animals. Finally, another limitation of the study is that we targeted a high-order area that is presumably still undergoing synaptic refinement during early adolescence, but only a systematic analysis of many brain regions can assess the full extent of the effects of chronic sleep loss.

Epidemiological studies consistently find that adolescents build a chronic sleep debt during the school days, which they are assumed to “repay” during the weekends by sleeping 1–2 hours longer (Wolfson and Carskadon, 1998; Roenneberg et al., 2007; Leger et al., 2012). Our protocol of chronic sleep restriction was quite severe but relatively short lasting (50–60% sleep loss for 5 days), but whether the milder but repeated pattern of sleep restriction observed in humans impairs the maturation of brain circuits is unknown. The inter-individual variability of the structural effects of chronic sleep loss in adolescents is also unknown, but adults vary in their susceptibility to the cognitive impairment caused by sleep deprivation (Van Dongen et al., 2004; Rupp et al., 2012), and differences in the microstructure of white and grey matter can predict inter-individual differences in the resistance to sleep loss (Rocklage et al., 2009; Cui et al., 2015; Bernardi et al., 2016).

We investigated the effect of gender in our study because sex differences in sleep exist in both humans and rodents (Mong et al., 2011): relative to males, adult female C57BL/6J mice (the strain used in the current study) are awake ∼1.5 hours more per day, recover relatively more sleep after acute sleep deprivation (Paul et al., 2006), and respond to restraint stress with a smaller rebound in REM sleep (Paul et al., 2009). Moreover, CSR mice were kept awake using mild forced locomotion, exposure to novel objects and social enrichment. None of these methods is routinely used in chronic variable stress paradigms. Yet, sleep is tightly homeostatically regulated and sleep pressure becomes irresistible even after just a few hours of extended wake (Borbely et al., 2016). Thus, extending wake beyond its physiological duration is inherently stressful, and chronic sleep loss in male adult rats leads to increased levels of catecholamines, and to a lesser extent, ACTH and glucocorticoids (Rechtschaffen and Bergmann, 2002). The behavioral effects of stress are sexually dimorphic. For instance, C57BL/6J mice that were kept awake by gentle handling for 3 hours daily from P5 to P42 show changes in sociability and repetitive behavior (but not in anxiety measures) when tested as adults, and these effects differ between males and females (Saré et al., 2016). The structural effects of chronic stress are also sexually dimorphic in rodent prefrontal cortex and hippocampus, with loss of dendritic spines only seen in males but not in females (Leuner and Shors, 2013), although the underlying mechanisms are unclear and may include different sensitivity to glucocorticoids (Gillies and McArthur, 2010; Leuner and Shors, 2013), and/or differences in the response to other hormones and neurotransmitters involved in stress and arousal, including glutamate or noradrenaline (Valentino et al., 2012). Because our two groups of mice included a similar number of males and females (14 males, 8 controls; 14 females, 6 controls) we specifically test for any sex-related difference in our findings, but could not find any. However, we cannot rule out that the number of animals may have been too small to detect subtle differences.

Loss of sleep is associated with cellular stress, impaired protein synthesis, and increased energy demand (Mackiewicz et al., 2008; Vecsey et al., 2012; Borbely et al., 2016; de Vivo et al., 2016), consistent with a general anabolic role for sleep. Although all mice gained weight between P21 (weaning) and P30 (end of CSR), controls did so more than CSR mice (gain weight in grams, C: +44.2 ± 12.2%; CSR: +20.7 ± 9.3%; *t* test, *p* < 0.001). Of note, in each group males gained more weight than females, almost twice as much in the CSR group, suggesting that CSR affected body growth more in females than males (Controls/F +34.8 ± 12.7%; Controls/M +51.1 ± 12.2%; *t* test, *p* = 0.007; CSR/F +15.8 ± 9.8%; CSR/M +27.9 ± 8.3%; *t* test, *p* = 0.008). Independent of the body however, growth and maintenance of neural circuits is energetically expensive and requires continuous protein synthesis (Kleim et al., 2003; Li et al., 2004). Of note, a recent study subjected flies to total sleep deprivation for 36 hours starting soon after eclosion and tested them as “young adults” (5 days old; Kayser et al., 2014). In these male flies, courtship behavior was impaired, and the volume of one specific olfactory glomerulus was reduced (Kayser et al., 2014). Intriguingly, this glomerulus was the one showing the largest growth after eclosion, suggesting that the most rapidly maturing brain regions are uniquely sensitive to sleep deprivation (Kayser et al., 2014). It is possible, therefore, that had we tested younger mice, we would have found more severe permanent structural effects caused by early chronic sleep loss.

## Synthesis

The decision was a result of the Reviewing Editor Rae Silver and the peer reviewers coming together and discussing their recommendations until a consensus was reached. A fact-based synthesis statement explaining their decision and outlining what is needed to prepare a revision is listed below. The following reviewer(s) agreed to reveal their identity: James Krueger

General comments

The manuscript investigates an important subject that many have opinions about but there is little data in support. That being that chronic sleep loss in adolescents is associated with long-term brain anatomical changes. This is a very well written and executed manuscript. There is a real possibility however, that the approach used to detect structural changes might have failed to detect any changes of any kind no matter what the perturbation. If the authors were to show a positive control, this would confirm that the approach works, and would confirm that the lack of structural change after CSR is a real effect. A positive control would add much to the paper and to conclusions that would be widely disseminated given the broad interest in the subject. Ideally, the authors would show in a brain region for which CSR (or sleep deprivation) has a known impact that this method can detect the change. If not, perhaps the method is not suited to draw any conclusions, and the absence of an effect could be methodological, not biological. This represents a substantial caveat."

Specific comments

1) Please indicate the number of times mice entered the water during deprivation; the ambient temperature (23 degrees)is well below thermoneutral temperatures for mice and if they were wet it would amplify the thermochallenge.

2) First half of the second paragraph of the Discussion - omit. Trying to make such comparisons between mice and men is pure speculation.
